# Outcomes and predictors of kidney failure in elderly patients with biopsy-proven IgA nephropathy

**DOI:** 10.1080/0886022X.2026.2629129

**Published:** 2026-02-19

**Authors:** Gabriel Ștefan, Nicoleta Petre, Adrian Zugravu, Simona Stancu

**Affiliations:** a Department of Nephrology, University of Medicine and Pharmacy “Carol Davila”, Bucharest, Romania; b Department of Nephrology, “Dr. Carol Davila” Teaching Hospital of Nephrology, Bucharest, Romania; c Department of Pathology, University of Medicine and Pharmacy “Carol Davila”, Bucharest, Romania; d Department of Pathology, “Dr. Carol Davila” Teaching Hospital of Nephrology, Bucharest, Romania

**Keywords:** IgA nephropathy, aged, chronic kidney disease progression, proteinuria, comorbidity, renin–angiotensin system inhibitors

## Abstract

The clinical presentation and prognosis of immunoglobulin A nephropathy (IgAN) in older adults are poorly defined. We evaluated kidney and patient outcomes and predictors of progression in patients aged ≥60 years with biopsy-proven IgAN. We conducted a retrospective observational study in elderly patients diagnosed with primary IgAN between 2010 and 2024 at a tertiary center. Baseline clinical, laboratory, and histopathologic data were collected, and kidney biopsies were scored using the Oxford MEST-C (Mesangial hypercellularity, Endocapillary hypercellularity, Segmental glomerulosclerosis, Tubular atrophy/interstitial fibrosis, Crescents) classification. The primary outcome was a composite of end-stage kidney disease (ESKD) or death. Predictors of progression were assessed using Kaplan–Meier’s analysis and Cox proportional hazards models. A total of 102 patients were included (median age 65 years; 73% male). Comorbidity burden was high (median Charlson Comorbidity Index 4), hypertension was prevalent (88%), and baseline kidney function was reduced (median estimated glomerular filtration rate (eGFR) 29.5 mL/min/1.73 m^2^). Chronic nephritic syndrome was the most frequent presentation (55%), and chronic histologic lesions predominated (T1/T2 in 52%, S1 in 32%). Over a median follow-up of 5 years, 49 patients (48%) reached the composite outcome, including 32 (31%) who progressed to ESKD. Older age, higher comorbidity burden, and greater proteinuria independently predicted progression, whereas baseline eGFR and individual MEST-C lesions did not. Renin–angiotensin system inhibitor use was associated with better outcomes, while no independent benefit of immunosuppression was observed after adjustment. Elderly patients with IgAN present with advanced chronic kidney disease and substantial comorbidity. Progression is driven mainly by age, comorbidity burden, and proteinuria, supporting optimized supportive care as the cornerstone of management.

## Clinical implications

- Elderly patients with IgA nephropathy (IgAN) frequently present with advanced chronic kidney disease (CKD), underscoring the need for timely referral and careful biopsy selection.
- Proteinuria remains the strongest modifiable predictor of progression in older adults and should be the primary therapeutic target irrespective of histologic findings.
- Comorbidity burden independently influences renal and patient outcomes, highlighting the importance of comprehensive geriatric and cardiovascular risk assessment.
- Oxford MEST-C lesions appear to have limited independent prognostic value in elderly patients with advanced disease, suggesting reduced utility for aggressive risk stratification.
- Renin–angiotensin system inhibition is associated with improved outcomes and should be optimized whenever tolerated in this population.
- Immunosuppressive therapy did not confer renal or survival benefit, supporting a cautious, individualized approach in elderly patients.
- High competing risks of cardiovascular death and infection should be explicitly considered when weighing immunosuppression versus supportive care.
- Clinical decision-making in elderly IgAN should prioritize functional status, comorbidities, and life expectancy over histologic activity alone.
- These findings support guideline recommendations favoring optimized supportive care rather than routine immunosuppression in older adults with IgAN.
- Future risk stratification models for IgAN should incorporate age and comorbidity indices to better reflect prognosis in elderly populations.

## GRAPHICAL ABSTRACT

**Figure:**
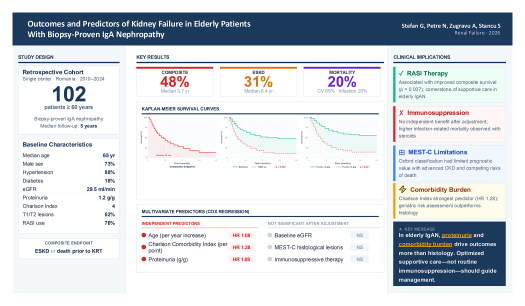

## Introduction

Immunoglobulin A nephropathy (IgAN), defined by dominant mesangial IgA deposition, is the most common primary glomerulopathy diagnosed by kidney biopsy and typically affects young adults [1–3]. Although 15–40% of patients progress to end-stage kidney disease (ESKD) within two decades, the condition becomes much less common in older individuals; in the United States, IgAN accounts for only ∼5% of biopsies in those older than 64 years, and in Europe the estimated annual incidence among individuals >60 years is as low as 0.3 per 100,000 [3,4].

Evidence on IgAN presentation, management, and outcomes in the elderly remains limited. Available studies suggest that older patients exhibit more hypertension, reduced kidney function, and greater chronic tubulointerstitial damage at biopsy than younger adults, and they experience faster kidney function decline and higher mortality [5]. These differences likely reflect age-related structural and functional renal changes that influence the clinical phenotype of IgAN. However, much of the existing evidence comes from small cohorts or biopsy registries with incomplete characterization of comorbidities, histopathology, and treatment exposure [6–10]. Geographical variation further complicates interpretation, as the incidence and clinical patterns of IgAN differ markedly across regions, with the highest burden reported in Asia [1]. Such disparities may also influence disease presentation and prognosis in elderly populations.

To address these gaps, we aimed to comprehensively evaluate kidney survival and its predictors in patients aged ≥60 years with biopsy-proven IgAN at a large tertiary nephrology center in Romania.

## Methods

### Study population

We conducted a retrospective observational study of patients with biopsy-proven primary IgAN diagnosed between January 2010 and October 2024 at a tertiary nephrology center in Romania. During this period, 520 patients were diagnosed with IgAN by kidney biopsy at our institution. Of these, 138 patients were aged ≥60 years at the time of kidney biopsy. Eligibility required a minimum follow-up duration of 12 months after kidney biopsy. Consequently, 36 patients were excluded: 20 due to secondary forms of IgAN or underlying systemic diseases (including IgA vasculitis, chronic liver disease, autoimmune disorders, or active malignancy), 10 because their follow-up duration was shorter than 12 months, and six due to incomplete clinical or histopathologic data. The remaining 102 patients fulfilled all inclusion criteria and constituted the final study cohort.

The study was conducted in accordance with the Declaration of Helsinki and was approved by the local Ethics Committee (Dr CD Teaching Hospital of Nephrology No. 32/2025), which waived the requirement for informed consent owing to the retrospective study design.

### Data collection and definitions

Clinical variables obtained from electronic medical records at the time of kidney biopsy included age, sex, Charlson Comorbidity Index, obesity (defined as a body mass index (BMI) > 30 kg/m^2^), diabetes mellitus, and arterial hypertension, which was defined as blood pressure > 140/90 mmHg or the use of antihypertensive medication [11]. Information on ongoing therapy with renin–angiotensin system inhibitors (RASIs) and the use of immunosuppressive medication was also collected. Laboratory data included serum creatinine, estimated glomerular filtration rate (eGFR) calculated using the Chronic Kidney Disease Epidemiology Collaboration (CKD-EPI) equation, proteinuria expressed as grams of protein per gram of urinary creatinine, and hematuria expressed as erythrocytes per high power field. The decision to initiate immunosuppressive therapy was at the discretion of the attending nephrologist.

Acute nephritic syndrome was defined as the sudden onset (duration < 3 months) of hematuria with dysmorphic red blood cells and/or red blood cell casts, associated with impaired kidney function, and variable degrees of proteinuria. Chronic nephritic syndrome was defined as persistent hematuria and/or proteinuria with a slowly progressive decline in kidney function and often long-standing hypertension, with a duration ≥3 months. Nephrotic syndrome was defined according to Kidney Disease: Improving Global Outcomes (KDIGO) criteria as proteinuria > 3.5 g/day or a protein-to-creatinine ratio ≥ 3 g/g, accompanied by hypoalbuminemia. Nephrotic–nephritic syndrome was defined by the coexistence of clinical and laboratory features characteristic of both nephrotic and nephritic presentations. Chronic kidney disease (CKD) was defined according to KDIGO guidelines as an eGFR < 60 mL/min/1.73 m^2^ and/or markers of kidney damage – such as persistent proteinuria or hematuria – for at least 3 months, in patients who did not meet the criteria for chronic nephritic syndrome or nephrotic syndrome, thereby ensuring that all diagnostic categories were mutually exclusive.

### Kidney biopsy

The diagnosis of IgAN was based on light microscopy, immunofluorescence (dominant IgA in the mesangium), and electron microscopy (para-mesangial electron-dense deposits). All the kidney biopsy specimens were reviewed by an experienced nephropathologist blinded to the clinical and laboratory data and were scored according to MEST-C (Mesangial hypercellularity, Endocapillary hypercellularity, Segmental glomerulosclerosis, Tubular atrophy/interstitial fibrosis, Crescents) Oxford Classification [12].

### Outcomes

The primary outcome was a composite endpoint consisting of (1) ESKD – defined as initiation of kidney replacement therapy (hemodialysis, peritoneal dialysis, or kidney transplantation) – or (2) death prior to the start of kidney replacement therapy. Patients were followed until they reached the composite endpoint or until 1 November 2025, whichever came first.

### Statistical analyses

Continuous variables were expressed as medians with interquartile ranges and compared between groups using the Mann–Whitney *U*-test. Categorical variables were summarized as frequencies and percentages and compared using the *χ*^2^ test or Fisher’s exact test, as appropriate.

Survival analyses were conducted to evaluate time to the composite endpoint, ESKD, and all-cause mortality. Kaplan–Meier’s curves were generated and compared using the log-rank test to assess differences in survival according to clinical, laboratory, and histologic parameters.

Cox proportional hazards regression models were used to identify factors associated with the composite endpoint. Variables with a *p* value <0.05 in univariate analysis were entered into the multivariate Cox model to determine independent predictors. Hazard ratios with 95% confidence intervals (CIs) were calculated for all Cox models. Statistical significance was set at a two-sided *p* value <0.05.

Analyses were performed using SPSS version 27 (Chicago, IL) and GraphPad Prism version 9.5.1 (GraphPad Software, La Jolla, CA).

## Results

### Baseline clinical and histological characteristics

In this cohort of 102 patients aged ≥60 years with biopsy-proven IgAN, the median age at diagnosis was 65 years, with a predominance of males (73%) and a high burden of comorbid conditions, reflected by a median Charlson Comorbidity Index of 4. Hypertension (88%) was highly prevalent, and chronic nephritic syndrome (55%) represented the most frequent clinical presentation, whereas acute nephritic (23%), nephrotic (10%), and nephrotic–nephritic (4%) syndromes were less common. Baseline kidney function was substantially reduced, with a median eGFR of 29.5 mL/min/1.73 m^2^ (IQR 18.8–43.0), and over half of the patients were already in CKD stage 3–5 at the time of biopsy. Median proteinuria was 1.2 g/g (0.7–2.9), with nephrotic-range proteinuria (>3 g/g) present in 26% of cases, and hematuria was observed in 91%. Patients were followed for a median of 5 years (95% CI 2.3–7.8).

Histopathologically, mesangial hypercellularity (M1) was documented in 45% of biopsies, endocapillary hypercellularity (E1) in 31%, and segmental glomerulosclerosis (S1) in 32%. Tubular atrophy and interstitial fibrosis (T1/T2) were present in 52% of patients, while crescents (C1/C2) were identified in 27%. RASIs were used in 74% of the cohort, and immunosuppressive therapy – most commonly corticosteroids – was administered in 63% (Table 1).

**Table 1.** Baseline characteristics of IgA nephropathy patients diagnosed at age ≥ 60 years.

	All *N* = 102	Composite endpoint		
		Yes *n* = 52	No *n* = 50	*p*
Age, years	65 (62, 69)	65 (63, 70)	65 (62, 68)	0.3
Male, *n* (%)	74 (73)	40 (77)	34 (68)	0.3
Obesity, *n* (%)	33 (33)	15 (29)	18 (37)	0.4
MAP, mmHg	100 (93.3, 106.6)	101.6 (93.3, 110)	96.6 (93.3, 103.3)	0.1
Charlson Comorbidity Index	4 (3, 5)	4 (3, 6)	4 (2, 4)	0.06
Hypertension, *n* (%)	90 (88)	45 (87)	45 (90)	0.5
Diabetes, *n* (%)	24 (24)	10 (19)	14 (28)	0.2
Clinical presentation, *n* (%)				<0.001
Acute nephritic syndrome	24 (23)	20 (39)	4 (8)	
Chronic nephritic syndrome	56 (55)	18 (35)	38 (76)	
Nephrotic syndrome	10 (10)	8 (15)	2 (4)	
Nephrotic-nephritic syndrome	4 (4)	2 (4)	2 (4)	
CKD	8 (8)	4 (7)	4 (8)	
eGFR, mL/min/1.73 m^2^	29.5 (18.3, 43)	24.9 (13.5, 37.7)	36.4 (26.3, 52)	0.002
CKD stage, *n* (%)				0.1
Stage 1/2	9 (9)	4 (7)	5 (10)	
Stage 3	41 (40)	16 (31)	25 (50)	
Stage 4	30 (29)	17 (33)	13 (26)	
Stage 5	22 (22)	15 (29)	7 (14)	
Proteinuria, g/g	1.2 (0.5, 2.7)	2 (0.9, 4.7)	0.9 (0.3, 1.7)	<0.001
P-uria >1 g/g, *n* (%)	60 (59)	37 (71)	23 (46)	0.01
Hematuria, *n* (%)	91 (89)	46 (89)	45 (90)	0.8
Hematuria, cells/HPF	185 (48, 250)	185 (43, 245)	190 (50, 250)	0.6
M1, *n* (%)	45 (44)	17 (33)	28 (56)	0.01
E1, *n* (%)	31 (30)	16 (31)	15 (30)	0.9
S1, *n* (%)	32 (31)	12 (23)	20 (40)	0.06
T1/2, *n* (%)	52 (51)	23 (44)	29 (58)	0.1
C1/2, *n* (%)	27 (27)	18 (35)	9 (18)	0.05
ACEI/ARB, *n* (%)	42 (41)	15 (29)	27 (54)	0.01
Immunosuppression, *n* (%)	64 (63)	37 (71)	27 (54)	0.07
Corticosteroid (gucococorticoid) therapy only	31 (30)	18 (35)	13 (26)	0.3
CFM	33 (33)	19 (37)	14 (28)	0.3

ACEI/ARB: angiotensin converting enzyme inhibitor/angiotensin receptor blocker; C1/2: crescents; E1: endothelial hypercellularity; eGFR: estimated glomerular filtration rate; ESKD: end-stage kidney disease; CFM: cyclophosphamide; CKD: chronic kidney disease; M1: mesangial hypercellularity; S1: segmental glomerulosclerosis; T1/2: tubular atrophy and interstitial fibrosis; synd: syndrome.

### Baseline differences by endpoint

A total of 52 patients (51%) reached the composite endpoint during follow-up (Table 1). Patients who reached the composite endpoint exhibited a more severe baseline renal profile, characterized by both lower eGFR (median 24.9 vs. 36.4 mL/min/1.73 m^2^, *p* = 0.002) and higher proteinuria (1.7 vs. 0.9 g/g, *p* < 0.001), indicating more advanced kidney dysfunction at presentation. Clinical presentation differed significantly between groups (*p* < 0.001), with acute nephritic syndrome being more frequent among patients who progressed to the composite endpoint (39% vs. 8%). Histologically, the distribution of MEST-C lesions was generally similar between groups, with the exception of crescentic lesions (C1/C2), which were more common in patients who reached the endpoint (18% vs. 9%, *p* = 0.05). Use of RASIs was lower in the endpoint group (54% vs. 74%, *p* = 0.01), while the proportion receiving immunosuppressive therapy was similar between groups (Table 1).

During follow-up, 32 patients (31%) progressed to ESKD, with a median renal survival of 8.4 (95% CI 4.8–11.9) years. Compared with those who did not progress, patients who reached ESKD had significantly worse baseline kidney function, including a lower median eGFR (22.5 vs. 35.5 mL/min/1.73 m^2^, *p* < 0.01) and higher proteinuria (2.1 vs. 0.9 g/g, *p* < 0.01). CKD stage distribution also showed a trend toward more advanced stages among patients who progressed (*p* = 0.06). There were no significant differences between groups in clinical presentation or in the distribution of MEST-C lesions, with the exception of mesangial hypercellularity (M1), which was less frequent in the ESKD group (25% vs. 53%, *p* = 0.009) (Supplementary Table 1).

Overall, 20 patients (20%) died during follow-up, with a mean patient survival of 10.2 (95% CI 8.9–11.5) years (mean reported because median survival was not reached). Those who died were older at diagnosis (68 vs. 65 years, *p* = 0.03) and had a higher Charlson Comorbidity Index (*p* = 0.01). Clinical presentation also differed between groups (*p* = 0.01), with deaths occurring more often in patients presenting with acute nephritic and nephrotic syndromes. Histologic findings were broadly comparable, although corticosteroid monotherapy was more common among patients who died (50% vs. 36%, *p* = 0.03). Deaths were most commonly attributable to cardiovascular disease (*n* = 13), with infections (*n* = 4) and neurological complications (*n* = 3) accounting for the remaining cases (Supplementary Table 1).

### Survival analysis and predictors of the composite endpoint

**Figure 1.**
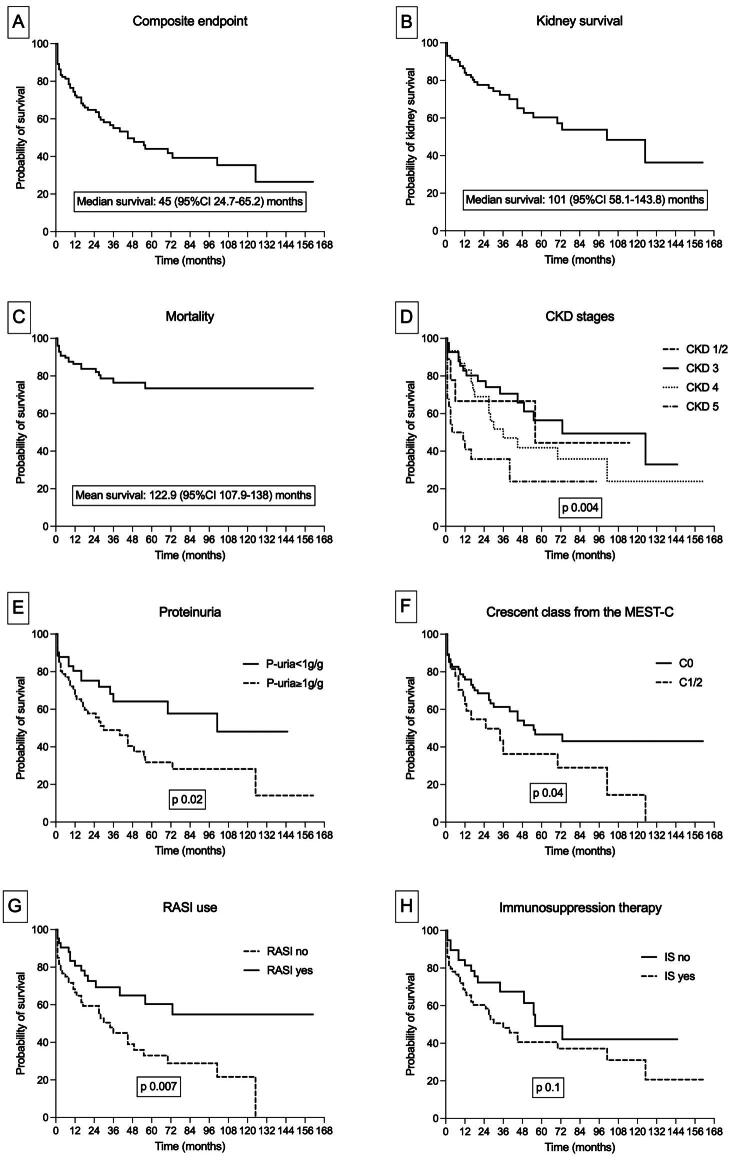
Kaplan–Meier’s survival analyses in elderly patients with IgA nephropathy. (A) Composite endpoint survival, defined as the time from biopsy to end-stage kidney disease (initiation of dialysis or kidney transplantation) or death prior to kidney replacement therapy. Median survival was 45 months (95% CI 24.7–65.2). (B) Kidney survival, defined as time to kidney failure. Median kidney survival was 101 months (95% CI 58.1–143.8). (C) Overall survival, showing time to all-cause mortality, with a mean survival of 122.9 months (95% CI 107.9–138). (D) Kidney survival stratified by CKD stage at the time of biopsy (CKD 1/2, 3, 4, 5), demonstrating significantly different survival curves (*p* = 0.004). (E) Event-free survival according to baseline proteinuria, comparing patients with proteinuria <1 g/g versus ≥1 g/g (*p* = 0.02). (F) Survival according to crescent class (MEST-C score), comparing C0 versus C1/2 lesions (*p* = 0.04). (G) Effect of renin–angiotensin system inhibitor (RASI) therapy on survival, showing improved outcomes in patients receiving RASI treatment (*p* = 0.007). (H) Survival according to immunosuppressive therapy, comparing patients who did versus did not receive immunosuppression (*p* = 0.1). All curves display probability of survival over follow-up time (months); *p* values derived from log-rank tests.

The median time to the composite endpoint was 3.7 (95% CI 2–5.4) years. In the Kaplan–Meier analysis, patients with advanced CKD stages, proteinuria >1 g/g, crescentic lesions (C1/2), and those not receiving angiotensin-converting enzyme inhibitor (ACEI)/angiotensin receptor blocker (ARB) therapy reached the composite endpoint more rapidly, whereas immunosuppressive therapy was not associated with differences in survival (Figure 1).

In the univariate Cox regression analysis, older age, higher Charlson Comorbidity Index, lower baseline eGFR, higher proteinuria, and the presence of crescents (C1/2) were associated with an increased risk of reaching the composite endpoint. In contrast, ACE inhibitor/ARB therapy was associated with a lower risk of progression (Table 2).

**Table 2.** Cox proportional hazard regression analysis examining predictors of composite endpoint in patients with IgA nephropathy diagnosed at age >60 years old.

	Univariate analysis		Multivariate analysis^a^	
	HR (95% CI)	*p*	95% CI	*p*
Age, per 1-year increase	1.07 (1.00–1.14)	0.02	1.08 (1.01–1.15)	0.02
Sex		0.5	–	
Male	Reference			
Female	0.82 (0.43–1.57)			
Obesity		0.9	–	
No	Reference			
Yes	1.01 (0.55–1.85)			
MAP, per 1-mmHg increase	1.00 (0.98–1.02)	0.6	–	
Charlson Comorbidity Index, per 1 point increase	1.30 (1.11–1.52)	<0.001	1.28 (1.07–1.53)	<0.01
Hypertension		0.2	–	
No	Reference			
Yes	0.62 (0.28–1.40)			
Diabetes		0.8	–	
No	Reference			
Yes	0.92 (0.46–1.86)			
eGFR, per 10-mL/min/1.73 m^2^ decrease	1.34 (1.12–1.61)	<0.01	1.01 (0.99–1.03)	0.1
Proteinuria, g/g	1.07 (1.00–1.15)	0.03	1.08 (1.00–1.17)	0.04
Mesangial hypercellularity		0.4	–	
M0	Reference			
M1	0.80 (0.44–1.44)			
Endothelial hypercellularity		0.1	–	
E0	Reference			
E1	1.57 (0.85–2.89)			
Segmental sclerosis		0.7	–	
S0	Reference			
S1	0.91 (0.47–1.75)			
Tubular atrophy/interstitial fibrosis		0.9	–	
T0	Reference			
T1/2	0.99 (0.56–1.73)			
Crescents		0.05		0.1
C0	Reference		Reference	
C1/2	1.75 (0.99–3.10)		1.54 (0.80–2.93)	
ACEI/ARB		0.01		0.1
No	Reference		Reference	
Yes	0.45 (0.24–0.83)		0.56 (0.27–1.13)	
Immunosuppression		0.1	–	
No	Reference			
Yes	1.58 (0.87–2.89)			

ACEI/ARB: angiotensin converting enzyme inhibitor/angiotensin receptor blocker; CI: confidence interval; eGFR: estimated glomerular filtration rate; HR: hazard ratio; MAP: mean arterial blood pressure.

^a^ Only statistically significant variables from univariate analysis were introduced in the multivariate model.

In the multivariate model, only older age, Charlson Comorbidity Index and proteinuria remained independently associated with the composite endpoint, while associations with eGFR, crescents, and RASI therapy were no longer statistically significant (Table 2). The loss of statistical significance for baseline eGFR in the multivariate model likely reflects collinearity with proteinuria and Charlson Comorbidity Index rather than biological irrelevance of kidney function, as these variables capture overlapping aspects of disease severity.

## Discussion

In this study of elderly patients with biopsy-proven IgAN, we found that most individuals presented with substantial comorbidity, advanced CKD, and predominantly chronic rather than active inflammatory lesions. Progression to ESKD or death was common – nearly half of the cohort reached the composite endpoint – and was strongly driven by baseline disease severity, particularly higher proteinuria and greater comorbidity burden. In contrast, the prognostic contribution of individual histopathologic features was comparatively limited. While RASI therapy was associated with improved outcomes, no independent benefit of immunosuppressive treatment was observed after adjustment. Mortality remained high and was mainly due to cardiovascular and infectious events, highlighting the complex interaction between kidney disease, frailty, and competing risks in this population.

Our results align closely with existing literature describing the distinct presentation and prognosis of IgAN in older adults (Table 3). Multiple studies have shown that elderly IgAN patients typically exhibit more comorbidities, higher prevalence of hypertension, more chronic tubulointerstitial damage, and more advanced CKD at diagnosis [7,10, 13,14]. In our cohort, the median eGFR at biopsy was markedly reduced (29.5 mL/min/1.73 m^2^), consistent with the advanced renal impairment reported in two recent U.S. series [10,14]. Similarly, the predominance of chronic nephritic syndrome and relatively infrequent nephrotic presentations mirrors observations in Chinese and Spanish elderly cohorts [8,9].

**Table 3.** Major studies on elderly IgAN patients.

Author, year	Country	Elderly definition (>years)	Size (*n*)	Age (years)	Male (%)	Hypertension (%)	eGFR (mL/min/1.73 m^2^)	Proteinuria (g/g or g/24 h)	M1 (%)	E1 (%)	S1 (%)	T1/2 (%)	C1/2 (%)	RASI (%)	IS (%)	ESKD (%)	Death (%)	Major findings
Frimat et al. [7]	France	50	33	61.6	82	66	39.9	3.1	6	n/a	50	72	44	n/a	n/a	18	n/a	Patients >50 had more impaired renal function and comorbidities; overall renal survival comparable to younger patients.
Wen and Chen [13]	Taiwan	65	17	71.9	65	88.2	25	3.1	n/a	n/a	n/a	n/a	23	n/a	n/a	22.2	44.4	Elderly patients more often presented with acute renal failure (52.9%) and nephrotic syndrome (41.2%), had more intercurrent infections and higher mortality, but similar renal survival compared with young adults.
Cheungpasitporn et al. [14]	USA (Mayo)	65	45	71	69	62	29	2.4	95	16	44	45	14	33	31	n/a	n/a	Elderly had higher comorbidity burden and more tubulointerstitial fibrosis/vascular sclerosis and showed faster progression to ESKD and higher mortality than younger adults.
Sevillano et al. [8]	Spain	65	151	72	77	n/a	32	2.1	66	28	37	51	n/a	84	46	26	20	In elderly, hematuria-related AKI was common; anticoagulation frequently present; outcomes poor with high risk of kidney replacement therapy or death.
Tan et al. [9]	China	50	126	57.1	52	27.8	66.5	2.7	79	8	57	17	23	n/a	n/a	10		Age, male sex, higher hematuria, S and T were independent risk factors for poor renal outcome; IS therapy did not improve outcomes over optimized RASI.
Zhang et al. [15]	China	60	182	64	58	79.1	46.1	1.8	64	40	38	56	61	n/a	n/a	n/a	n/a	Elderly patients had higher proteinuria, lower eGFR, more HTA, more severe E and less severe S; tubular/interstitial p16INK4a positivity correlated with T, eGFR, and proteinuria.
Rizk et al. [10]	USA	60	127	69.4	73	71	25	81% >1 g/g	24	13	43	22	15	n/a	n/a	35	68	Elderly cohort with high comorbidity and advanced CKD; 35% progressed to kidney failure over median 3.4 years and mortality was high; male sex and advanced CKD stage were key predictors of kidney failure, while older age and male sex predicted death.
Current study	Romania	60	102	65	73	88	29.5	1.2	44	30	31	51	27	41	63	31	20	Advanced CKD, high comorbidity, and proteinuria predicted progression; MEST-C lesions were not independently prognostic; RASI use was protective, and immunosuppression showed no clear benefit.

AKI: acute kidney injury; C1/2: crescents; CFM: cyclophosphamide; CKD: chronic kidney disease; E1: endothelial hypercellularity; eGFR: estimated glomerular filtration rate; ESKD: end-stage kidney disease; HTA: arterial hypertension; IS: immunosuppression; M1: mesangial hypercellularity; RASI: renin angiotensin system inhibitors; S1: segmental glomerulosclerosis; T1/2: tubular atrophy and interstitial fibrosis; USA: United States of America.

### Proteinuria and CKD stage as dominant prognostic factors

In line with the broader IgAN literature, proteinuria emerged as a central determinant of progression (Table 3). Elderly cohorts in both Asian and Western populations consistently demonstrate that proteinuria is a key driver of renal decline [9,10,13]. Our multivariable model confirmed that higher baseline proteinuria remained independently associated with the composite endpoint, reinforcing its importance as a therapeutic target even in older patients.

Similarly, advanced CKD stage at presentation – common in our cohort – was strongly associated with poorer outcomes, consistent with prior studies showing that reduced eGFR is one of the most powerful prognostic markers in elderly IgAN [10,14]. Earlier evidence, including a 2013 systematic review, also suggests that older age may further increase vulnerability to progression, with older patients experiencing a higher risk of ESKD than their younger counterparts, although the included studies predated current biopsy practices and treatment strategies [5]. Together, these findings support the concept that age-related renal changes – such as nephrosclerosis, diminished renal reserve, and chronic low-grade inflammation – may amplify the impact of traditional risk factors in older adults

### Role of comorbidities and competing risks

One of the defining aspects of elderly IgAN is the influence of comorbidities on outcomes. The Charlson Comorbidity Index was independently associated with progression in our multivariable analysis, underscoring the interplay between systemic health and kidney disease trajectory. Similar observations were reported by Cheungpasitporn et al. who found that higher comorbidity burden was linked to worse renal and patient survival in elderly IgAN [14]. In the single-center U.S. cohort described by Rizk et al. diabetes and hypertension were highly prevalent, and advanced CKD stage and older age at diagnosis were major predictors of kidney failure and mortality, further emphasizing the contribution of non-disease-specific factors to prognosis in this population [10].

Mortality in our study (20%) was clinically significant, with cardiovascular disease and infections being the leading causes of death. These patterns replicate previous observations, underscoring that nonrenal death is a major competing risk in elderly IgAN and may modify the apparent effect of histopathologic predictors [8,10,13].

Future research in IgAN among the elderly should prioritize prospective, multicenter studies that integrate geriatric-specific factors such as frailty indices, functional status, and competing cardiovascular risks into prognostic models, as current evidence indicates that age, comorbidity burden, and proteinuria outweigh histopathologic lesions in determining outcomes. There is a critical need to refine risk stratification tools that move beyond traditional MEST-C scoring to incorporate biomarkers of chronicity, vascular injury, systemic inflammation, and complement activation, together with longitudinal changes in proteinuria and renal function.

### Therapeutic considerations: renin–angiotensin system (RAS) blockade and immunosuppression

Renin–angiotensin system blockade was associated with improved outcomes in univariate analyses in our cohort and in multiple previous studies [9,10]. This reinforces its role as foundational therapy even in elderly patients with advanced CKD, provided it is tolerated.

By contrast, immunosuppressive therapy did not significantly influence survival or renal progression. This finding is consistent with emerging evidence that the benefits of immunosuppression may be attenuated – or outweighed by risks – in older adults. Tan et al. found no clear advantage of immunosuppressive treatment over optimized supportive care in elderly patients, and earlier studies noted higher infection-related complications in this age group [9,13]. Our finding that steroid monotherapy was more common among deceased patients should be interpreted with caution, as it likely reflects confounding by indication in a non-randomized setting, but it supports the need for prudence when considering immunosuppression in elderly patients.

Randomized controlled trials specifically designed for older patients are warranted to evaluate the efficacy and safety of emerging therapies, including SGLT2 inhibitors, endothelin receptor antagonists, targeted-release budesonide, and complement pathway inhibitors such as agents targeting factor B, factor D, C3, or C5a signaling, compared with optimized supportive care, given the limited benefit and potential harm of conventional immunosuppression in this population. Particular emphasis should be placed on identifying biomarkers that predict response to complement-targeted therapies and on carefully assessing infection risk and other age-related adverse events. Additionally, future studies should formally account for competing risks of death and explore patient-centered outcomes, including quality of life and treatment burden, to better inform individualized, age-appropriate management strategies in elderly patients with IgAN.

### Crescents and MEST-C lesions in the elderly

Histologically, our cohort showed a high burden of chronic injury, with more than half of patients exhibiting T1/T2 lesions, consistent with prior reports of age-related tubulointerstitial damage [6,15]. Crescents were present in 27% of cases – similar to other elderly IgAN series, where C1/C2 lesions are less frequent than in younger adults but remain clinically relevant [9,10]. In our study, crescents were associated with progression in univariate analysis but lost significance after adjustment, suggesting that their prognostic weight is diluted by competing risks of death and the predominance of advanced CKD with extensive chronic damage. In this context, the high burden of comorbidity and cardiovascular mortality competes with kidney-specific endpoints, effectively attenuating the apparent effect of active histologic lesions such as crescents on renal progression.

Interestingly, mesangial hypercellularity (M1) was less common among patients who progressed to ESKD, a finding also noted in some elderly cohorts [9]. This pattern may reflect a shift from active inflammatory lesions toward chronic sclerotic changes in older individuals, in whom T lesions, vascular injury, and low baseline eGFR appear to be the dominant drivers of outcome [5,15].

### Strengths and limitations

The strengths of this study include a well-defined biopsy-proven elderly IgAN cohort, long follow-up duration, and detailed clinical and histopathological characterization. However, several limitations merit consideration. The retrospective single-center design limits causal inference, and treatment decisions – especially regarding immunosuppression – were not protocolized, introducing confounding by indication. Some pathologic features, including crescent subclasses and chronicity indices, were limited by sample size. As with all elderly IgAN cohorts, competing risks from comorbidity may obscure the kidney-specific prognostic effects of some histologic lesions. Finally, although we compared our findings with several contemporary cohorts, the heterogeneity in definitions of ‘elderly,’ treatment strategies, and biopsy thresholds across studies complicates direct comparison.

## Conclusions

In summary, elderly patients with IgAN frequently present with advanced CKD, substantial comorbidity, and chronic histopathologic injury. In this population, proteinuria and comorbidity burden were the dominant predictors of kidney disease progression, while the prognostic significance of individual MEST-C lesions – particularly crescents – was attenuated after adjustment. Renin–angiotensin system blockade was associated with improved outcomes, whereas immunosuppressive therapy did not clearly modify prognosis. These findings reinforce the idea of optimized supportive care and careful individualized decision-making regarding immunosuppression in older adults with IgAN.

## Supplementary Material

Supplemetnary_IgAN_elderly.docx

## Data Availability

The data underlying this article will be shared on reasonable request to the corresponding author.
