# Supplementary material for: Outcomes and predictors of kidney failure in elderly patients with biopsy-proven IgA nephropathy
Source: Ren Fail. 2026 Feb 19;48(1):2629129. doi: 10.1080/0886022X.2026.2629129 (PMC12922409; doi:10.1080/0886022X.2026.2629129)
Supplement: Supplemetnary_IgAN_elderly.docx [file IRNF_A_2629129_SM8093.docx]

|  | ESKD | | | Mortality | | |
| --- | --- | --- | --- | --- | --- | --- |
|  | Yes  n=32 | No  n=70 | p | Dead  n=20 | Alive  n=82 | p |
| Age, years | 65 (62, 68) | 66 (62, 69) | 0.4 | 68 (64, 72) | 65 (62, 68) | 0.03 |
| Male, % | 78 | 70 | 0.3 | 75 | 72 | 0.7 |
| Obesity, % | 41 | 29 | 0.2 | 11 | 38 | 0.06 |
| MAP, mmHg | 105 (96.6, 110) | 96.6 (93.3, 106.6) | 0.03 | 95 (90, 110) | 100 (93.3, 106.6) | 0.4 |
| Charlson score | 4 (3, 5) | 4 (2, 5) | 0.1 | 4 (3, 6) | 4 (2, 5) | 0.4 |
| Hypertension, % | 84 | 90 | 0.4 | 90 | 88 | 0.7 |
| Diabetes, % | 19 | 26 | 0.4 | 20 | 24 | 0.6 |
| Clinical presentation, %  Acute nephritic synd  Chronic nephritic synd  Nephrotic synd  Nephrotic-nephritic synd  CKD | 33  38  13  3  13 | 18  63  9  4  6 | 0.1 | 45  30  20  5  0 | 18  61  7  4  10 | 0.01 |
| eGFR, mL/min | 22.5 (11.7, 31.3) | 35.5 (23.3, 51.5) | <0.01 | 30.6 (17.4, 42.1) | 29.5 (19.7, 44.0) | 0.8 |
| CKD stage, %  Stage 1/2  Stage 3  Stage 4  Stage 5 | 0  31  34  35 | 13  44  27  16 | 0.06 | 20  30  30  20 | 6  43  29  22 | 0.2 |
| Proteinuria, g/g | 2 (1.3, 5) | 0.9 (0.4, 2.4) | <0.01 | 1.8 (0.5, 3.2) | 1.2 (0.5, 2.6) | 0.6 |
| P-uria >1g/g, % | 81 | 49 | 0.002 | 55 | 60 | 0.6 |
| Hematuria, % | 88 | 90 | 0.7 | 90 | 89 | 0.9 |
| Hematuria, cells/HPF | 195 (37, 250) | 180 (50, 250) | 0.9 | 165 (43, 220) | 195 (50, 250) | 0.4 |
| M1, % | 25 | 53 | 0.009 | 45 | 44 | 0.9 |
| E1, % | 28 | 31 | 0.7 | 35 | 29 | 0.6 |
| S1, % | 22 | 36 | 0.1 | 25 | 33 | 0.4 |
| T1/2, % | 47 | 53 | 0.5 | 40 | 54 | 0.2 |
| C1/2, % | 34 | 23 | 0.2 | 35 | 24 | 0.3 |
| ACEI/ARB, % | 28 | 47 | 0.07 | 30 | 44 | 0.2 |
| Immunosuppression, %  Corticotherapy only  CFM | 69  25  44 | 60  33  27 | 0.3  0.4  0.09 | 75  50  25 | 60  26  34 | 0.2  0.03  0.4 |
| ACEI/ARB, angiotensin converting enzyme inhibitor/angiotensin receptor blocker; C1/2, crescents; E1, endothelial hypercellularity; eGFR, estimated glomerular filtration rate; ESKD, end stage kidney disease; CFM, cyclophosphamide; CKD, chronic kidney disease; M1, mesangial hypercellularity; S1, segmental glomerulosclerosis; synd, syndrome; T1/2, tubular atrophy and interstitial fibrosis | | | | | | |

**Baseline characteristics of IgA nephropathy patiens diagnosed at age ≥ 60 years**
